# MRI of wrist ligament trauma was similar at 7 T and 3 T with arthroscopy as a reference standard

**DOI:** 10.1007/s00330-025-11656-4

**Published:** 2025-05-08

**Authors:** Simon Götestrand, Magnus Flondell, Björn Lundin, Elena Aksyuk, Rami Abu Shalhoub, Pawel Szaro, Erik Hedström, Anders Björkman, Mats Geijer

**Affiliations:** 1 Diagnostic Radiology, Department of Clinical Sciences Lund, Lund University, Lund, Sweden; 2 Department of Radiology, Skåne University Hospital, Lund, Sweden; 3 Department of Hand Surgery, Skåne University Hospital, Malmö, Sweden; 4 Department of Hand Surgery, Translational Medicine, Lund University, Malmö, Sweden; 5 Department of Radiology, Institute of Clinical Sciences, Sahlgrenska Academy, University of Gothenburg, Gothenburg, Sweden; 6 Department of Radiology, Region Västra Götaland, Sahlgrenska University Hospital, Gothenburg, Sweden; 7 Clinical Physiology, Department of Clinical Sciences Lund, Lund University, Lund, Sweden; 8 Department of Clinical Physiology, Skåne University Hospital, Lund, Sweden; 9 Department of Hand Surgery, Institute of Clinical Sciences, Sahlgrenska Academy, University of Gothenburg, Gothenburg, Sweden; 10 Department of Hand Surgery, Sahlgrenska University Hospital, Gothenburg, Sweden

**Keywords:** Imaging (Magnetic Resonance), Articular Ligaments, Triangular Fibrocartilage Complex, Injuries (Wrist), Arthroscopies

## Abstract

**Objectives:**

The aim was to compare the diagnostic accuracy of magnetic resonance imaging (MRI) at 7 Tesla (T) and 3 T in patients with clinically suspected injury of the triangular fibrocartilage complex (TFCC) or the scapholunate ligament (SLL) using wrist arthroscopy as a reference standard.

**Methods:**

Twenty-four patients scheduled for wrist arthroscopy due to suspected TFCC or SLL injury were examined with 7-T and 3-T MRI before arthroscopy. Four musculoskeletal radiologists independently evaluated the MR examinations in randomized order, and the findings were compared to those at wrist arthroscopy.

**Results:**

Sensitivity, specificity, and accuracy for TFCC injuries were 0.85, 0.68, and 0.82 for 7 T and 0.75, 0.73, and 0.77 for 3 T, respectively. For SLL injuries, the corresponding values were 0.70, 0.65, and 0.74 for 7 T and 0.69, 0.55, and 0.70 for 3 T, respectively. For both TFCC and SLL injuries, no statistically significant difference between 7 T and 3 T was found, and the confidence intervals for accuracy overlapped (0.67–0.94 vs 0.63–0.88 for TFCC injuries and 0.59–0.89 vs 0.52–0.86 for SLL injuries). In 14 of 24 patients (58%), MRI contributed findings of additional types of injuries, e.g., tendon injuries and ganglia, vital for a complete diagnosis.

**Conclusions:**

The diagnostic accuracy of MRI at 7 T was similar to 3 T for detecting injury to the TFCC and the SLL. A majority of injuries were correctly diagnosed by MRI, but some injuries found using arthroscopy were missed.

**Key Points:**

***Question*** *Previous studies have found that MRI cannot reliably diagnose or rule out TFCC or SLL injury, compared to the current reference standard wrist arthroscopy*.

***Findings*** *The diagnostic accuracy for MRI at 7 T was similar to 3 T for detecting injuries to the TFCC and the SLL*.

***Clinical relevance*** *Although MRI cannot replace wrist arthroscopy, it is an important complementary tool in the diagnostic workup of suspected wrist ligament injuries, with the ability to diagnose additional types of pathologies not accessible by arthroscopy*.

**Graphical Abstract:**

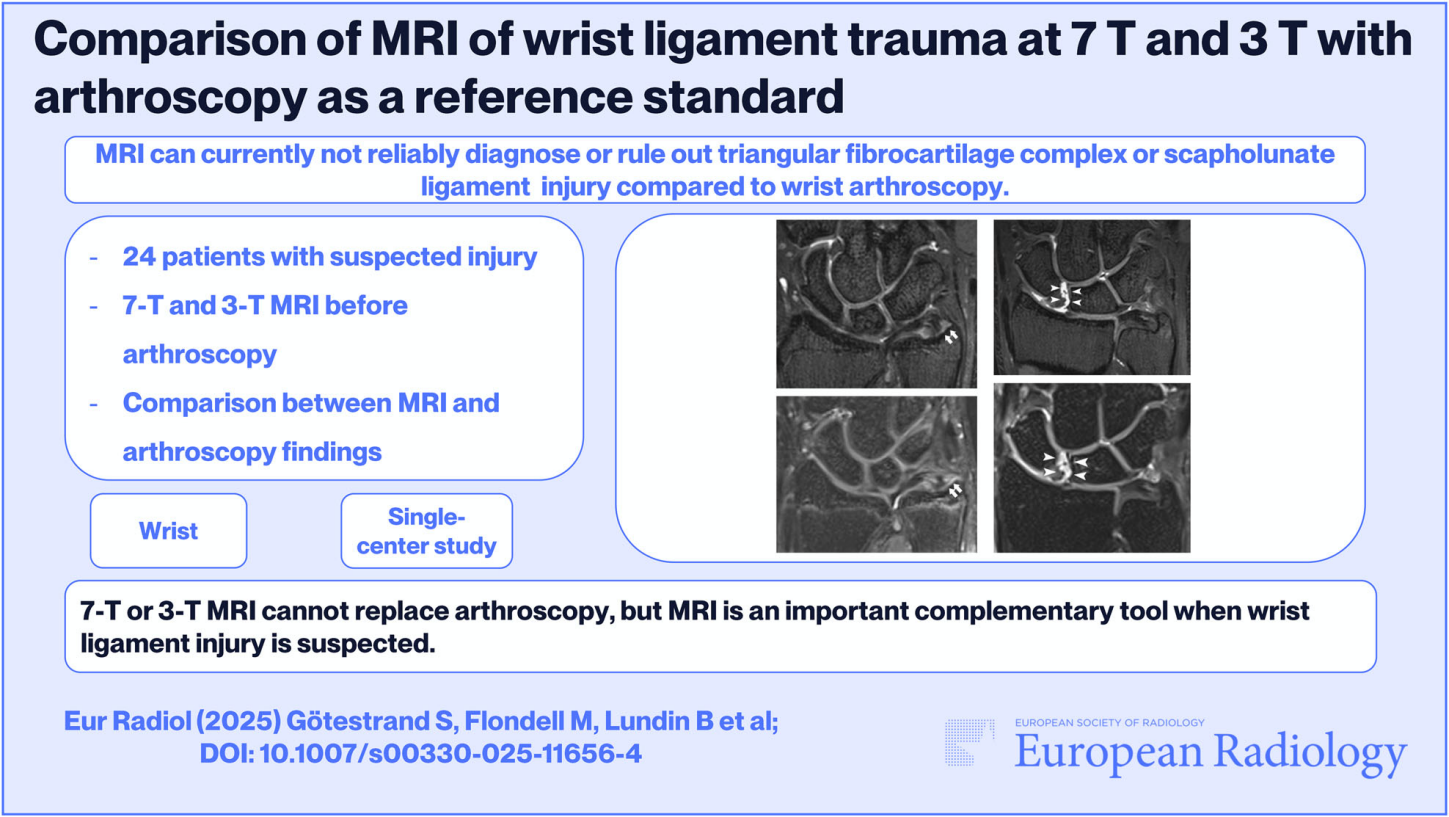

## Introduction

Wrist ligament injuries are common [1, 2] and may lead to pain, instability, and osteoarthritis if left unattended [3, 4]. Numerous ligaments stabilize the wrist, and the most important are the triangular fibrocartilage complex (TFCC) and the scapholunate ligament (SLL) [5, 6].

The anatomical complexity and small size of the TFCC and SLL make diagnostic imaging difficult [7, 8]. Therefore, many patients with wrist pain undergo wrist arthroscopy, which is the current reference standard for diagnosing ligament injuries [6]. Arthroscopy allows for immediate treatment if a ligament injury is detected. However, arthroscopy is costly and has a risk of complications. Improved non-invasive imaging could benefit both patients and the healthcare system, but the use of MRI is limited due to a lack of consensus on the effectiveness of MRI in evaluating wrist ligament injuries [6–8].

In a recent study, MRI at 7 Tesla (T) was found to improve visualization of the TFCC, the SLL, and the lunotriquetral ligament (LTL) in healthy individuals compared to 3 T imaging [9]. However, Heiss et al found 3-T MRI superior to 7-T in depicting the TFCC, the SLL, and the LTL in both healthy individuals and patients with chronic wrist pain [10]. These studies only investigated the visualization of anatomical structures and not diagnostic accuracy, as MRI findings were not correlated with arthroscopy findings. The diagnostic accuracy of 7-T MRI compared to arthroscopy in suspected wrist ligament injury has not yet been investigated.

The aims of this study were to investigate (1) if the diagnostic accuracy is higher for 7-T MRI than for 3-T MRI for suspected injury of the TFCC or the SLL, and (2) if TFCC or SLL injury can be ruled out with MRI at 7 T or 3 T, using wrist arthroscopy as a reference standard.

## Materials and methods

### Study design

The local ethics committee approved this single-center prospective study (2017/193), which was carried out in accordance with the Declaration of Helsinki. All patients received oral and written information about the study and gave written informed consent before participation.

### Participants

Patients scheduled for wrist arthroscopy due to a suspected TFCC or SLL injury between May 2020 and April 2024 were invited to participate in the study. Inclusion criteria were clinical signs of TFCC or SLL injury as determined by one of three expert-level hand surgeons, as defined by Tang et al [11]. Exclusion criteria were contraindications to MRI, previous surgical intervention in the wrist, inability to understand written or spoken instructions in Swedish, and an age below 18 years.

### MR imaging

Imaging was performed using a 7-T MR scanner (Achieva 7 T; Philips) with a single-channel transmit and a 16-channel receive wrist coil (RAPID Biomedical) and a 3-T MR scanner (Magnetom Vida Fit; Siemens Healthineers) with a 16-channel receive wrist coil (Siemens Healthineers). The 7-T and 3-T examinations were performed on the same day for each patient. The order of the examinations was determined according to the availability of the two scanners. The patients were positioned on the side, with the hand and wrist facing down, in a neutral position above the head.

The MRI protocols at 7 T and 3 T have been used in a previous study [9], and are detailed in Supplementary Tables 1 and 2. The operating hand surgeon received a radiology report before surgery, based on the findings from both the 7-T and 3-T examinations, written in consensus by two observers with 9 years (S.G.) and 30 (M.G.) years of experience with wrist MRI. These observers did not participate in the image evaluation in the study.

### Image evaluation

All images were pseudonymized, and the examinations were put in a randomized order, regardless of field strength, for assessment by four blinded observers. The order was different for each observer, and the 7-T and 3-T examinations for each subject were not assessed in conjunction. The observers had 35 years, 9 years, 5 years, and 3 years of experience as musculoskeletal radiologists. Before image evaluation, the observers participated in a tutorial session to ensure conformity in image assessment. Image evaluation for the 24 patients was carried out in two batches. The first batch consisted of 12 patients. Six of the patients from the first batch were also included in the second batch, for the calculation of intra-observer agreement. Thus, the second batch consisted of 18 patients. In cases where images were evaluated twice, the second assessment was used only for the calculation of intra-observer agreement. In addition to the 48 examinations included in this study (one at 7 T and one at 3 T for each patient), four additional examinations at 7 T and 3 T from two healthy participants from a previous study [9] were included to increase the number of cases without injury.

For the TFCC, the clinical grading system by Palmer [12] was used as a basis for evaluation, with updates and revisions of the Palmer classification taken into account [13–15]. Thus, the evaluation comprised the radial, styloid, and foveal parts of the TFCC, the ulnotriquetral and ulnolunar ligaments, as well as the dorsal and volar radioulnar ligaments and the ulnomeniscal homolog. Each structure was graded according to a five-grade scale. Grade 0: no injury, defined as a clearly delineated structure with low signal on all sequences; Grade 1: probably no injury, defined as partially increased signal intensity interpreted as edema in the structure but with preserved distinctness; Grade 2: possible injury defined as increased signal intensity interpreted as edema in the structure, also being indistinct; Grade 3: partial rupture of the structure, defined as a visual discontinuation of ligament fibers through part of the structure; and Grade 4: complete rupture of the structure, defined as a visual discontinuation of ligament fibers through the entire structure.

For the SLL, the dorsal and volar components of the ligament complex were evaluated separately. The proximal membranous component was not graded as it is principally composed of fibrocartilage, and is, as such, not a true ligament [16]. A four-grade scale was used. Grade 0: intact ligament, defined as a clearly delineated structure with low signal; Grade 1: increased signal intensity, interpreted as edema in, or indistinctness of, the structure; Grade 2: a partial or total rupture of the dorsal or volar component of the ligament, defined as a visual discontinuation of ligament fibers through part of, or the entire structure; Grade 3: rupture of both the dorsal and volar component of the ligament, with or without diastasis between the scaphoid and the lunate bones.

For further analysis, the findings were dichotomized to facilitate comparison with arthroscopy findings and to give a more clinically relevant interpretation (i.e., injury or no injury). For the TFCC, Grades 2–4 were considered an injury. For the SLL, Grades 1–3 were considered an injury.

The observers were also instructed to record pathologic findings in other wrist structures, such as tendon injuries or tenosynovitis, or ganglia.

### Wrist arthroscopy

Wrist arthroscopy was performed by one of three expert-level [11] arthroscopists. Forearm distraction was used (Vertical Traction Tower©, Håndkirurgi ApS). Under the tourniquet, standard 3–4, 4–5, and 6R portals were used to evaluate the radiocarpal joint. Midcarpal ulnar and radial portals were used to evaluate the midcarpal joint surfaces, the SLL, and the LTL. In the radial compartment, the SLL and the joint surfaces were inspected, and signs of injury were classified according to Geissler [17]. In the ulnar compartment, the TFCC was examined, and lesions were classified according to Palmer [12]. Additionally, the posterior capsule and underlying extrinsic volar ligaments were inspected. Testing for stability was done using an arthroscopic hook, and provocation tests included trampoline-, suction-, and hook tests.

### Statistics

For demographic data, median (range) and numbers (%) are presented. The MRI findings of the four observers at 7 T and 3 T were classified as true positive, false positive, true negative, and false negative, with findings at wrist arthroscopy as a reference standard. Sensitivity, specificity, positive predictive value, and negative predictive value were calculated.

A visual grading characteristics (VGC) analysis was done using the VGC analyzer software [18]. The resulting area under the curve (AUC) with the corresponding confidence interval was used to obtain a measure of the accuracy of MRI compared to wrist arthroscopy. The AUC was obtained using the observers’ evaluations of the arthroscopically negative cases as reference modality and the observers’ evaluations of the arthroscopically positive cases as test modality. VGC analysis was also done to compare the findings at 7 T and 3 T. The AUC was obtained using the observers’ evaluations of arthroscopically positive cases at 3 T as reference modality, using the observers’ evaluations of the arthroscopically positive cases at 7 T as test modality. The same analysis was done for arthroscopically negative cases. For all analyses, fixed-reader analysis was used, based on the trapezoid VGC curve.

Inter-observer and intra-observer agreement were evaluated by intraclass correlation coefficients (ICC) with 95% confidence intervals, using the dichotomized data as input and calculated using R [19]. For inter-observer agreement, the calculations were based on a mean-rating, agreement, two-way random-effects model. For intra-observer agreement, the calculations were based on a mean-rating, agreement, two-way mixed-effects model. Values less than 0.50 are suggested to indicate poor reliability; values between 0.50 and 0.75, moderate reliability; values between 0.75 and 0.90, good reliability; and values greater than 0.90, excellent reliability [20].

## Results

Twenty-four participants (7 women and 17 men) with a median age of 34 years (range 18–57 years) were included. The median interval between MRI and arthroscopy was 12 days (range 1–36 days). MRI at 7 T was performed before MRI at 3 T in seven (29%) cases. At arthroscopy, seven (29%) patients had a TFCC injury and 10 (42%) had an SLL injury.

**Fig. 1:**
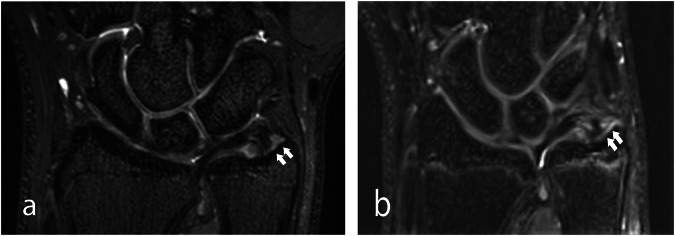
A 19-year-old male with right-sided wrist pain after a sports injury. 3D PD-weighted MRI showed a clearly delineated TFCC injury at the ulnar styloid (arrows) on coronal reformations at both (**a**) 7 T and (**b**) 3 T. TFCC, triangular fibrocartilage complex; T, Tesla

In all 26 subjects, the four observers found TFCC injury with MRI in 6, 11, 13, and 15 patients at 7 T, and in 7, 9, 12, and 12 patients at 3 T, respectively. An example of a TFCC injury is shown in Fig. 1. The ICC for interobserver agreement was 0.59 (95% CI 0.26–0.79) for 7 T and 0.74 (95% CI 0.53–0.87) for 3 T. The four observers correctly identified 4–7 of the 7 arthroscopically verified TFCC injuries at 7 T and 4–6 at 3 T, and in all, 82% were correctly identified at 7 T and 75% at 3 T (Table 1).

**Table 1:** Ranges of the number of injuries found on MRI, injuries found on arthroscopy, ranges of true positive, true negative, false positive and false negative, as well as sensitivity, specificity, positive predictive value, negative predictive value, and AUC with 95% confidence interval for TFCC and SLL injury at 7 T and 3 T

Structure	Field strength	Injury on MRI	Injury on arthroscopy	TP	TN	FP	FN	Sensitivity	Specificity	PPV	NPV	AUC	95% CI (for all observers)	*p* value
TFCC	7 T	6–15	7 (29%)	4–7	10–16	2–9	0–2	0.85 (0.67–1.00)	0.68 (0.53–0.89)	0.49 (0.44–0.67)	0.93 (0.89–1.00)	0.82	0.67–0.94	0.0005
TFCC	3 T	7–12	7 (29%)	4–6	12–15	3–7	1–3	0.75 (0.57–0.86)	0.73 (0.63–0.83)	0.51 (0.46–0.57)	0.88 (0.83–0.92)	0.77	0.63–0.88	0.004
SLL	7 T	6–19	10 (42%)	5–10	6–15	1–9	0–5	0.70 (0.50–1.00)	0.65 (0.40–0.94)	0.56 (0.43–0.83)	0.77 (0.67–1.00)	0.74	0.59–0.89	0.006
SLL	3 T	8–20	10 (42%)	5–10	6–13	3–10	0–5	0.69 (0.50–1.00)	0.55 (0.38–0.81)	0.48 (0.42–0.63)	0.74 (0.64–1.00)	0.70	0.52–0.86	0.02

*AUC* area under the curve, *CI* confidence interval, *FN* false negative, *FP* false positive, *MRI* magnetic resonance imaging, *NPV* negative predictive value, *PPV* positive predictive value, *SLL* scapholunate ligament, *T* Tesla, *TFCC* triangular fibrocartilage complex, *TN* true negative, *TP* true positive

**Fig. 2:**
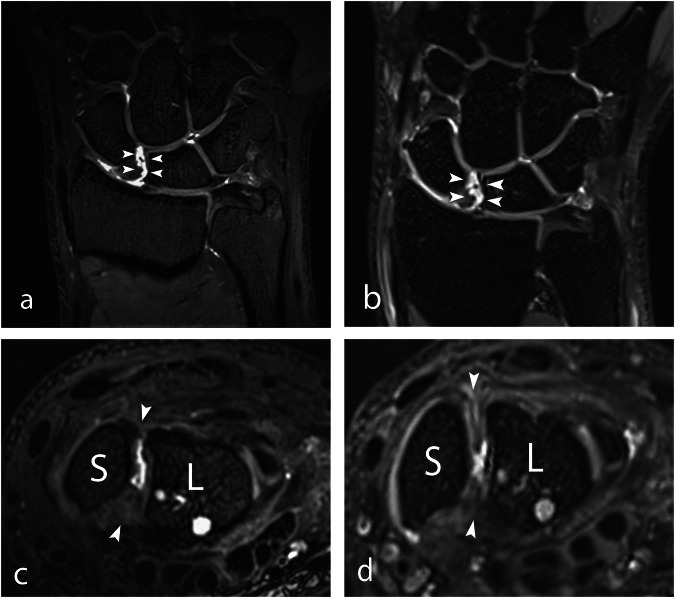
A 24-year-old male with right-sided wrist pain after a work-related injury. 3D PD-weighted MRI showed a clearly delineated SLL injury (arrowheads) on coronal and axial reformations at both (**a**, **c**) 7 T and (**b**, **d**) 3 T. SLL, scapholunate ligament; T, Tesla; S, scaphoid; L, lunate

**Fig. 3:**
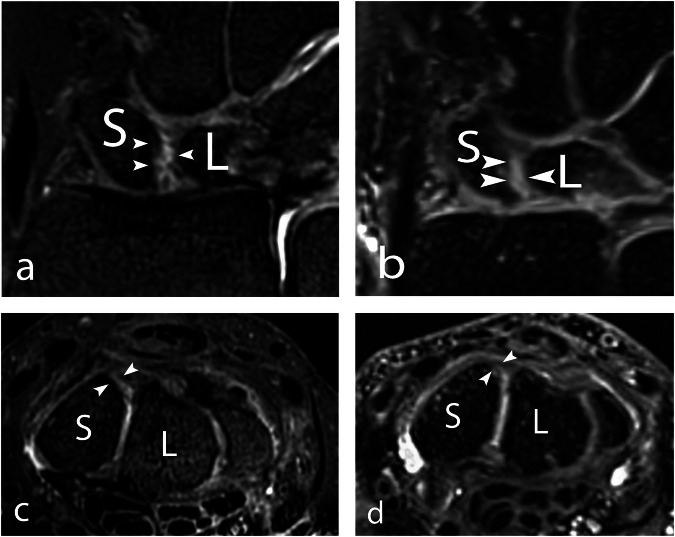
A 52-year-old male with left-sided wrist pain after a work-related injury. A subtle dorsal SLL injury (arrowheads) can be discerned on coronal and axial reformations at (**a**, **c**) 7-T and (**b**, **d**) 3-T 3D PD-weighted MRI. Multiplanar reformation was necessary to visualize this injury. SLL, scapholunate ligament; T, Tesla; S, scaphoid; L, lunate

**Fig. 4:**
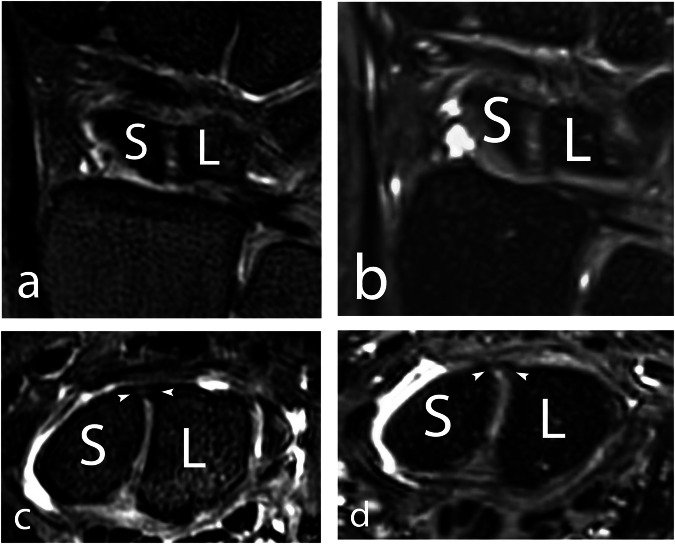
A 44-year-old male with an intact dorsal component of the SLL on 7-T and 3-T MRI. The dorsal component of the SLL (arrowheads) can be clearly delineated on coronal and axial reformations at (**a**, **c**) 7-T and (**b**, **d**) 3-T 3D PD-weighted MRI. SLL, scapholunate ligament; T, Tesla; S, scaphoid; L, lunate

In the evaluation of SLL injuries, injuries were found in 6, 11, 14, and 19 patients at 7 T, and in 8, 12, 16, and 20 patients at 3 T, respectively. Figure 2 shows an example of an SLL rupture, and Fig. 3 demonstrates a more subtle rupture of the dorsal part of the SLL. Figure 4 shows an intact dorsal part of the SLL for comparison. The interobserver agreement calculated by ICC was moderate: 0.63 (95% CI 0.32–0.82) for 7 T and 0.69 (95% CI 0.43–0.85) for 3 T. Of the 10 arthroscopically verified SLL injuries, the four observers identified 5–10 at both 7 T and 3 T, and in all, 70% were correctly identified at 7 T and 68% at 3 T (Table 1).

Table 1 shows the number of TFCC and SLL injuries found at arthroscopy, along with the range of injuries found at MRI by the four observers and the range of true positive, true negative, false positive, and false negative findings. The sensitivity, specificity, positive predictive value, negative predictive value, and AUC values with 95% confidence intervals, as well as *p* values for TFCC and SLL injuries, are also reported. No statistically significant difference was found between 7 T and 3 T compared to arthroscopic findings in the evaluation of injury of the TFCC or the SLL (Table 2).

**Table 2:** VGC analysis, comparing the findings at 7 T and 3 T

Structure	Injury on arthroscopy	AUC	95% CI (for all observers)	*p* value
TFCC	Yes	0.53	0.31–0.72	0.81
TFCC	No	0.48	0.36–0.59	0.67
SLL	Yes	0.49	0.27–0.71	0.93
SLL	No	0.44	0.34–0.54	0.29

The MRI findings in patients with arthroscopically verified injury, of TFCC and SLL, respectively, are compared. Also, the MRI findings in patients where no injury was found at arthroscopy, at TFCC, and SLL, respectively, are compared. An AUC_VGC_ of 0.5 shows that the findings, on average, are equal at 7 T and 3 T. An AUC_VGC_ of > 0.5 shows that the findings correlated better with arthroscopy at 7 T. An AUC_VGC_ of < 0.5 shows that the findings correlated better with arthroscopy at 3 T. If there is a statistically significant difference between grading, the confidence interval does not contain 0.5

*AUC* area under the curve, *CI* confidence interval, *MRI* magnetic resonance imaging, *SLL* scapholunate ligament, *T* Tesla, *TFCC* triangular fibrocartilage complex, *VGC* visual grading characteristics

The arthroscopy findings for each of the 24 patients with the corresponding gradings from each observer are detailed in Supplementary Table 3. The symptom duration is also detailed. There was no correlation between experience in musculoskeletal radiology and agreement with arthroscopy findings, or the number of false-positive gradings. False positive gradings were randomly distributed between observers, as well as patients.

By MRI, several other findings contributing to posttraumatic wrist pain were found. Two were tendon rupture (one with complete rupture of the extensor carpi radialis brevis tendon and one with split rupture of the extensor carpi ulnaris tendon), six were tenosynovitis, and 12 were wrist ganglia. All these findings were visible at both field strengths.

Intra-observer agreement was poor to excellent across the four observers. Intra-observer ICC was for observer 1: 0.74 (95% CI 0.40–0.89); for observer 2: 0.67 (95% CI 0.23–0.86); for observer 3: 0.86 (95% CI 0.44–0.85); and for observer 4: 1 (thereby no CI). For observer 4, a post hoc analysis showed that this perfect match was related to the data dichotomization, whereas the underlying grading was slightly different between repeated assessments.

## Discussion

The current study showed no clear advantage of MRI at 7 T over 3 T for evaluation of TFCC or SLL injuries. Although most injuries were identified by MRI, the accuracy of MRI at 7 T for correctly identifying injuries was not higher than previously achieved at lower field strengths [21, 22].

Arthroscopy is considered the reference standard to diagnose TFCC and SLL injuries. However, it is a technically demanding procedure that requires more resources than imaging and has a complication rate of almost 5% [23]. A non-invasive imaging modality capable of identifying or excluding TFCC and SLL injuries would limit the need for diagnostic arthroscopy. So far, the accuracy of MRI in diagnosing or excluding TFCC or SLL injury is too low to replace wrist arthroscopy. However, both the current and previous studies [21, 22] show that a majority of TFCC and SLL injuries can be detected by MRI. In the current study, the range of sensitivity and specificity was somewhat narrower between observers for both TFCC and SLL injuries than in a systematic review [22], where MRI had a sensitivity and specificity for detecting TFCC lesions that ranged between 0.44–0.93 and 0.54–1.00, respectively, and correspondingly for SLL lesions 0.11–0.89 and 0.55–1.00. No measurements of accuracy were provided in that study. Further, in the current study, 82% and 75% of TFCC injuries and 70% and 68% of SLL injuries were correctly identified at 7 T and 3 T, respectively, compared to the accuracy of 0.89–0.91 for detecting TFCC injuries and 0.75–0.92 for detecting SLL injuries in a systematic review [21].

The inter-observer agreement was poor to good for both 7 T and 3 T. The varying inter-observer agreements found are likely related to the varying experience levels between observers. Alternatively, it may reflect the difficulty in evaluating small wrist ligaments, as great variation has also previously been reported [21, 22]. The wide range of intra-observer agreement found in the current study is in line with these hypotheses. The inter- and intra-observer agreements highlight the necessity of multiple observers in studies such as this, as the results otherwise may be misleading, or at least not generalizable.

Diagnostic accuracy was not higher for 7 T than for 3 T. This is despite that 7 T previously has shown superior visualization of TFCC and the SLL compared to 3 T in healthy individuals [9]. This exemplifies that it may be more difficult to delineate injuries to these structures than to visualize them anatomically with MRI. It also points to the general importance in radiology to assess diagnostic accuracy, and not only image quality. A tear through a small ligament may be subtle. Especially when no bone malalignment is present, a torn ligament may still lie in its anatomical position, with the tear only being discernible when tension is exerted on the ligament. Also, an increased water signal due to edema may obscure or potentially simulate a lesion.

3 T MRI has been shown to improve the diagnostic performance in detecting TFCC and SLL injury compared to 1.5 T [24, 25]. As the current study shows, further increasing the field strength to 7 T did not alleviate the challenges of depicting injuries to wrist ligaments using MRI, and additional aspects of image acquisition could be investigated. For example, if the SLL is torn, an MRI with the fist clenched may increase the separation between the torn ligament ends, potentially sharpening the diagnosis. Ultrasound allows for rapid, cost-effective, and dynamic evaluation of wrist ligaments, and may be an under-utilized modality [26]. Carpal ligaments can be visualized with ultrasound, and injury should be suspected if the ligament fibers are discontinuous or if the ligament is not seen in its expected anatomic location [27]. Using dynamic maneuvers, injuries can be seen [26]. Finally, MR arthrography at 7 T can be utilized, as it has been shown to better depict wrist ligament injuries than conventional MRI at lower field strengths [21, 25, 28, 29]. However, MR arthrography is still an invasive procedure, albeit less demanding for the patients, and with a lower risk of complications compared to arthroscopy.

Although the current study shows that most injuries of the TFCC and the SLL are correctly identified by MRI, some are missed. Whether the accuracy of a diagnostic test reaches the threshold level of being clinically useful depends on many factors, such as the cost, availability, and risk of complications associated with the reference standard [30]. The findings of the current study suggest that MRI complements wrist arthroscopy rather than serving as its replacement, and in selected cases, it may be possible to confirm or rule out suspected ligament injury. As is also shown, MRI is capable of demonstrating pathologies undiagnosed by wrist arthroscopy, such as traumatic tendon injuries and tenosynovitis. Degenerative conditions, osteonecrosis, osteomyelitis, arthritis, tumors, and injury to wrist ligaments not accessible using arthroscopy can also be diagnosed with MRI [31]. MRI findings may also give direction to the surgeon, showing the location and severity of pathologies. A close collaboration between the radiologist and the hand surgeon is important to maximize the gains from pre-operative MRI, where the radiologist must have a thorough understanding of which pathologies the hand surgeon needs to know about before arthroscopy, and clearly communicate these findings.

A limitation of this study was that the sample selection was likely biased to a high likelihood of injury, since the patients were scheduled for arthroscopy. Another limitation was the small number of patients. In part, the impact of this on the results was decreased by the prospective inclusion and collaboration with the same three hand surgeons, the same-day MR examinations at both 7 T and 3 T, and the participation of four blinded MRI readers with various degrees of expertise, which also augment the generalizability of the results.

## Conclusions

MRI at 7 T showed no clear advantage over 3 T imaging for the evaluation of injuries to the TFCC and the SLL using wrist arthroscopy as a reference. Neither MRI at 7 T nor 3 T can replace wrist arthroscopy, but MRI is an important complementary tool in the diagnostic workup of suspected wrist ligament injuries, with the ability to diagnose additional types of pathologies not accessible with arthroscopy.

## Supplementary information

ELECTRONIC SUPPLEMENTARY MATERIAL

## Abbreviations

AUC: Area under the curve
LTL: Lunotriquetral ligament
MR: Magnetic resonance
MRI: Magnetic resonance imaging
SLL: scapholunate ligament
T: Tesla
TFCC: Triangular fibrocartilage complex
VGC: Visual grading characteristics
