# Supplementary material for: MRI of wrist ligament trauma was similar at 7 T and 3 T with arthroscopy as a reference standard
Source: Eur Radiol. 2025 May 8;35(11):6949–57. doi: 10.1007/s00330-025-11656-4 (PMC12559061; doi:10.1007/s00330-025-11656-4)
Supplement: Supplementary file 1 — ELECTRONIC SUPPLEMENTARY MATERIAL [file 330_2025_11656_MOESM1_ESM.pdf]

MRI of wrist ligament trauma was similar at 7T and 3T with arthroscopy as a reference standard

ELECTRONIC SUPPLEMENTARY MATERIAL

Eur Radiol (2025) Götestrand S, Flondell M, Lundin B, et al.

Supplementary Table 1. Parameters for the 7 T wrist MR protocol

	2D protocol				3D
	PD TSE			T1 TSE	PD TSE
**Plane of acquisition**	axial	coronal	sagittal	coronal	coronal
**Repetition time** (ms)	1816	2963	2544	300	1100
**Matrix size**	200x225	200x225	200x166	300x300	184x181
**Echo time** (ms)	28	28	28	8.8	110
**Turbo factor** (echo train length)	7	7	7	3	22
**Receiver bandwidth** (Hz/pixel)	324	324	306	503	534
**Field of view** (mm^2)	70x81	70x81	70x50	89x89	82x82
**Reconstructed voxel dimensions** (mm^3)	0.24x0.24x1.2	0.24x0.24x1.2	0.22x0.22x1.2	0.28x0.28x2.0	0.24x0.24x0.24
**Aquired voxel dimensions** (mm^3)	0.35x0.36x1.2	0.35x0.36x1.2	0.35x0.36x1.2	0.30x0.30x2.0	0.45x0.45x0.50
**Number of slices**	30	30	42	15	240
**Phase oversampling**	No	No	No	No	No
**Slice oversampling**	N/A	N/A	N/A	N/A	10%
**In-plane phase encoding direction**	RL	RL	AP	RL	RL

**Fat saturation**	Yes	Yes	Yes	No	Yes
**Partial Fourier faith sampling**	No	No	No	7/10	No
**Acceleration technique**	SENSE 1.4	SENSE 1.4	SENSE 1.4	None	SENSE 2.2
**Acquisition time** (min:sec)	3:02	4:50	3:08	2:11	7:07
**Driven Equilibrium**	No	No	No	No	Yes

AP, anteroposterior; MR, magnetic resonance; N/A, not applicable; PD, proton density; RL, right-left; SPACE, "Sampling Perfection with Application optimized Contrasts using different flip angle Evolution"; TSE, turbo spin-echo

Supplementary Table 2. Parameters for the 3 T wrist MR protocol

	Clinical 2D protocol				3D
	PD TSE			T1 TSE	PD SPACE (GRE)
**Plane of acquisition**	axial	coronal	sagittal	coronal	coronal
**Repetition time** (ms)	5580	4070	5050	846	1100
**Matrix size** (frequency encoding steps x phase encoding steps)	320x240	320x240	320x240	320x256	448x306
**Echo time** (ms)	26	24	26	25	39
**Turbo factor** (echo train length)	13	13	13	3	29
**Receiver bandwidth** (Hz/pixel)	223	223	223	233	385
**Field of view** (mm^2)	80x80	90x90	80x80	100x100	220x150
**Reconstructed voxel dimensions** (mm^3)	0.3x0.3x2	0.3x0.3x2	0.3x0.3x3	0.3x0.3x2	0.5x0.5x0.5
**Number of slices**	30	22	25	15	112
**Phase oversampling**	40%	40%	40%	0%	25%
**Slice oversampling**	N/A	N/A	N/A	N/A	29%

**In-plane phase encoding direction**	AP	RL	AP	RL	RL
**Fat saturation**	Yes	Yes	Yes	No	Yes
**Interpolation**	No	No	No	No	No
**Partial Fourier faith sampling**	No	No	No	No	7/8
**Acceleration technique**	GRAPPA 2	None	GRAPPA 2	None	GRAPPA 2
**Acquisition time** (min:sec)	3:45	5:27	2:23	4:53	7:05

AP, anteroposterior; GRE, gradient-echo; MR, magnetic resonance; N/A, not applicable; PD, proton density; RL, right-left; SPACE, "Sampling Perfection with Application optimized Contrasts using different flip angle Evolution"; TSE, turbo spin-echo

Supplementary Table 3. Wrist arthroscopy findings for 24 patients referred to arthroscopy for suspicion of TFCC or SLL injury with corresponding gradings at MRI scoring given by each of the four observers. The grades in agreement with arthroscopic findings are presented in bold. The duration of symptoms for each patient is detailed. The respective years of experience in musculoskeletal radiology, the percentage of gradings in agreement with arthroscopy findings and the number of false positive gradings for each observer are also listed.

Patient	Symptoms (months)	Tear at arthroscopy		Observer 1 (7 T/3 T)		Observer 2 (7 T/3 T)		Observer 3 (7 T/3 T)		Observer 4 (7 T/3 T)	
		TFCC	SLL	TFCC	SLL	TFCC	SLL	TFCC	SLL	TFCC	SLL
1	>12	Yes	No	**3/3**	1/1	**2/2**	**0/0**	**4/4**	**0/0**	**3**/1	2/2
2	3-12	Yes	No	**2/3**	**0**/1	1/1	**0**/2	**2/3**	**0/0**	**2/3**	**0/0**
3	>12	Yes	No	**4**/1	1/**0**	**3**/0	2/1	**2/2**	**0/0**	**4/4**	1/2
4	>12	No	Yes	3/**0**	**3/3**	**1/1**	**3/3**	2/**1**	**3/3**	2/**1**	**3/3**
5	1-3	No	Yes	**1/1**	**3/1**	3/3	**3/3**	**0/1**	**3/3**	2/**1**	**2/2**
6	>12	No	No	3/3	2/1	**0/0**	**0/0**	2/2	**0**/1	**1/0**	2/2
7	>12	No	No	**1**/3	2/1	**0**/3	**0**/2	**1**/2	**0/0**	2/3	2/2
8	3-12	No	No	**0**/3	**0/0**	**0/1**	**0**/1	2/3	**0/0**	**0**/3	**0/0**
9	>12	No	No	**1**/2	1/1	3/**1**	**0**/1	**1/1**	**0/0**	2/**1**	1/1
10	>12	No	No	2/**1**	1/2	**1/0**	**0/0**	2/**1**	**0/0**	2/**1**	2/**0**
11	>12	Yes	No	**2/2**	**0/0**	1/**3**	**0/0**	1/1	**0/0**	**3/3**	**0/0**
12	3-12	Yes	Yes	**3/3**	0/**1**	**3/2**	**1/2**	**4/4**	0/0	**4/4**	**2/2**
13	3-12	No	No	2/**1**	1/**0**	**0/0**	**0/0**	**0/0**	**0/0**	**0/0**	2/2

14	>12	No	No	**1/1**	**0**/1	**1/1**	**0**/2	**0/0**	**0**/1	**0/0**	1/2
15	>12	Yes	No	**3/2**	**0/0**	**3/3**	**0/0**	**2/2**	**0/0**	**3/3**	**0/0**
16	>12	No	Yes	2/**1**	0/0	**0/0**	**1**/0	**0/0**	0/0	**0**/2	**2/2**
17	3-12	No	Yes	**1/1**	**1**/0	**1/1**	**1/1**	**0**/2	0/0	**1/1**	**2/2**
18	3-12	No	Yes	2/3	**3/3**	3/**1**	**2/3**	**1/0**	**2/3**	**1/0**	**3/3**
19	>12	No	Yes	**1/1**	0/0	**1/1**	0/0	**1/1**	0/0	2/**0**	**1/1**
20	>12	No	No	2/**1**	1/1	**1/1**	1/1	**0/1**	**0**/1	**0/1**	1/2
21	3-12	No	Yes	3/3	**3/3**	**1**/2	**3/3**	2/2	**3/3**	3/4	**3/3**
22	3-12	No	Yes	**1**/2	**1/1**	**1/0**	**2/2**	**0/1**	**2/1**	**0/1**	**2/3**
23	>12	No	Yes	2/**1**	0/0	**1/1**	0/0	**0/0**	0/0	**1/1**	**1/1**
24	>12	Yes	No	**2/2**	**0/0**	0/1	1/1	**2/2**	**0/0**	0/0	1/2
Years of observer experience				35 years		9 years		5 years		3 years	
Percentage of gradings in agreement with arthroscopy findings (7T/3T)				63%/ 67%	50%/ 50%	75%/ 75%	79%/ 54%	75%/ 75%	79%/ 67%	67%/ 75%	58%/ 63%
Number of false positive gradings (7 T/3 T)				9/7	8/8	3/3	3/8	5/5	0/3	7/4	10/9

MRI, magnetic resonance imaging; SLL, scapholunate ligament; T, Tesla; TFCC, triangular fibrocartilage complex.
